# Magnetic Resonance Elastography for Staging Liver Fibrosis in the Oncopig

**DOI:** 10.3390/diagnostics14171880

**Published:** 2024-08-28

**Authors:** Ron C. Gaba, Lobna Elkhadragy, Thomas Pennix, Kyle M. Schachtschneider, Courtni R. Bolt, Aaron Anderson, Shreyan Majumdar, Denise Weber, Herbert E. Whiteley, Daniel P. Regan, Lawrence B. Schook, Bradley P. Sutton

**Affiliations:** 1 Department of Radiology, University of Illinois at Chicago, Chicago, IL 60612, USA; lobna@uic.edu (L.E.); schook@illinois.edu (L.B.S.); 2 Department of Radiology, University of Texas, Houston, TX 77058, USA; 3 Sus Clinicals Inc., Cincinnati, OH 45242, USA; 4 Department of Animal Sciences, University of Illinois at Urbana-Champaign, Champaign, IL 61801, USA; 5 Beckman Institute for Advanced Science & Technology, University of Illinois at Urbana-Champaign, Champaign, IL 61801, USAbsutton@illinois.edu (B.P.S.); 6 Department of Bioengineering, University of Illinois at Urbana-Champaign, Champaign, IL 61801, USA; 7 College of Veterinary Medicine, University of Illinois at Urbana-Champaign, Champaign, IL 61801, USA; herb.whiteley@gmail.com; 8 Flint Animal Cancer Center, Colorado State University, Fort Collins, CO 80523, USA; dn.regan@gmail.com

**Keywords:** swine, liver, fibrosis, liver cirrhosis, magnetic resonance imaging

## Abstract

This pilot study investigated the feasibility of using magnetic resonance elastography (MRE) for the non-invasive detection and quantification of liver fibrosis in the Oncopig cancer model. Seven 8-week-old Oncopigs underwent alcoholic liver fibrosis induction and serial MRE imaging and liver biopsy at 1, 2, and 3 months post procedure. MRE was utilized to quantify liver stiffness, and liver fibrosis was histologically graded using the METAVIR system. The primary outcome measure was the capability to detect and quantify liver fibrosis using MRE with radiologic–pathologic correlation. Liver fibrosis induction, MRE imaging, and liver biopsy were successfully performed. MRE liver fibrosis was evident in 57% (4/7), 50% (3/6), and 40% (2/5) animal subjects 1, 2, and 3 months after fibrosis induction, with mean liver stiffness of 2.94, 3.25, and 2.91 kPa, respectively. Histological liver fibrosis was noted in 71% (5/7), 100% (5/5), and 100% (5/5) of animal subjects with available tissue samples. There was no significant statistical correlation between the MRE-measured liver stiffness and the METAVIR fibrosis scores. In conclusion, quantifiable liver fibrosis may be induced in the Oncopig. MRE has potential utility in non-invasively detecting liver stiffness in this large-animal preclinical model, though tissue biopsy was more sensitive in demonstrating disease.

## 1. Introduction

Liver cirrhosis is a leading cause of morbidity and mortality worldwide, accounting for nearly 2 million deaths per year [1,2]. It represents an advanced stage of liver fibrosis and is characterized by distortion of the hepatic architecture due to excessive accumulation of extracellular matrix. Major risk factors for liver fibrosis include excessive alcohol consumption, chronic viral hepatitis, and metabolic dysfunction-associated steatotic liver disease (MASLD). Cirrhosis from any etiology is the strongest risk factor for the development of primary liver cancer, or hepatocellular carcinoma (HCC), with over 90% of cases arising in the context of chronic liver disease [3]. The global rise in liver fibrosis and HCC risk factors—including MASLD, which affects 25% of the global population [4]—highlights the significance of preclinical animal models that recapitulate human disease and allow the development and testing of novel diagnostic, imaging, and therapeutic strategies.

The Oncopig is an inducible transgenic porcine tumorigenic platform that has shown utility in the modeling of HCC for preclinical investigation of diagnostics, drugs, and devices [5,6,7]. Liver tumor development in the Oncopig can be achieved in a background of comorbid alcoholic liver fibrosis [8,9,10], an important comorbidity to model since alcoholic liver disease can incite liver cirrhosis, increase the risk for development of primary liver cancer, and have profound effects on HCC tumor biology and response to treatment [11]. To this end, the successful development and detection of liver fibrosis in the Oncopig is critical to ensuring the fidelity of this model in recapitulating human disease and expanding its utilization in preclinical studies. While liver fibrosis is typically diagnosed using invasive tissue biopsy, magnetic resonance imaging (MRI) and elastography (MRE) may provide a non-invasive portrayal of liver disease presence and severity [12]. The purpose of this pilot study was to investigate the feasibility of MRE for the non-invasive detection and quantification of Oncopig liver fibrosis.

## 2. Materials and Methods

### 2.1. Animal Subjects

**Figure 1:**
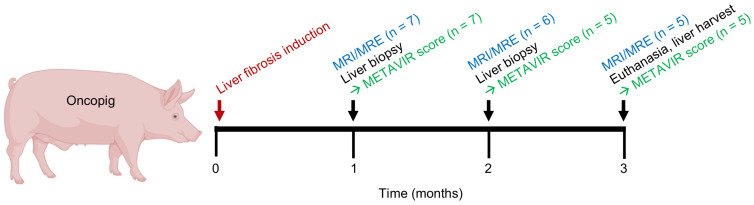
Graphical representation of experimental design. Of 7 experimental pigs, all underwent liver fibrosis induction, 1-month MRI/MRE, and 1-month biopsy. At 2 months, 6 pigs underwent MRI/MRE, and liver biopsy analysis was performed for 5 subjects. At 3 months, 5 pigs underwent MRI/MRE, and liver tissue analysis was performed for 5 subjects. Figure created with BioRender.com.

This study was performed at the University of Illinois at Urbana-Champaign under approval from the Institutional Animal Care and Use Committee. All animal procedures were performed in accordance with accepted animal welfare standards in a facility that is accredited by the American Association for Accreditation of Laboratory Animal Care (AAALAC) and the Office of Laboratory Animal Welfare (OLAW) of the National Institutes of Health, compliant with the United States Department of Agriculture Animal Welfare Act and Public Health Service Policy on the Humane Care and Use of Laboratory Animals. Two healthy 5-week-old female Oncopigs were initially used to test and optimize the MRI/MRE imaging protocol. Seven 8-week-old Oncopigs (six males, one female; mean weight 22 kg) were then enrolled as experimental subjects who underwent alcohol-based liver fibrosis induction, serial radiologic imaging, and successive biopsy procedures. A graphical representation of the experimental design is presented in Figure 1. The selected Oncopig cohort size and total number of radiologic exams for this pilot study reflected sample sizes typically used in exploratory pilot studies [13].

### 2.2. Liver Fibrosis Induction

With animal subjects under general anesthesia, alcoholic liver disease was generated by infusing an emulsion of absolute ethanol-ethiodized oil (Lipiodol Ultra Fluid; Guerbet, Villepinte, France) into the hepatic artery [8]. For this procedure, ultrasound-guided vascular access was gained into the common femoral artery with insertion of a 5-French sheath (Pinnacle; Terumo, Somerset, NJ, USA). Using standard catheter and wire techniques, a 2.7-French microcatheter was advanced into the gastroduodenal artery. This vessel was occluded with metallic coils (Nester Embolization Coil; Cook, Bloomington, IN, USA) to protect non-target ethanol administration into intestinal vessels. The microcatheter was then positioned in the proper hepatic artery, and 0.75 mL/kg of a 1:3 *v*/*v* emulsified mixture of absolute ethanol and ethiodized oil [8,14] was slowly infused into the hepatic arterial circulation over 30–45 min. Upon completion of the ethanol and ethiodized oil infusion, all devices were removed, and hemostasis was achieved with 10 min of manual compression at the vascular access site.

### 2.3. MRI/MRE

MRI/MRE was performed at 1, 2, and 3 months after liver fibrosis induction (pigs aged 3, 4, and 5 months), with measurement of liver parenchymal stiffness. The imaging protocol was made as compatible as possible with modern clinical liver imaging protocols. All scanning was completed on a Siemens 3T Prisma scanner using an 18-channel body coil and a 32-channel spine coil, with animal subjects under general anesthesia. The MRI/MRE protocol (Table 1) was configured to sample a broad range of sequences to capture the range of liver imaging performed clinically, including T1, T2, fat/water Dixon methods, diffusion-weighted imaging, and multiphase (arterial, venous, delayed) dynamic contrast-enhanced (DCE) imaging. It also took advantage of sequences that are robust to motion (e.g., balanced steady-state free precession line acquisition with undersampling, or BLADE) and respiratory-gated sequences. Respiratory gating removed the need for breath hold and captured the liver in the same position in each scan. For respiratory gating, a standard human respiratory belt was used to identify the acquisition window at end expiration. For DCE scans, intravenous gadodiamide (Omniscan; GE Healthcare, Chicago, IL, USA) (0.6 mL/kg bodyweight) was injected (120 mL/min) using an MRI-compatible pump (Harvard Apparatus) followed by saline flush.

MRE data were acquired to measure liver stiffness using a custom spin-echo (SE) echo-planar imaging (EPI) MRE sequence [15,16] with spatial resolution 4.9 × 4.9 × 5.0 mm, 12 slices, field of view 41.3 × 30.5 cm, and 60 Hz vibration encoded at 4 time points in 3 directions with positive and negative polarity for each direction. The sensitivity of the MRE encoding was 8.927 μm/rad. This acquisition was carried out inside a breath hold, where the ventilation of the pig was controlled during the breath hold. Actuation was applied at 60 Hz using a Resoundant mechanical actuator (Rochester, MN, USA). The liver was manually masked using ITK-snap (www.itksnap.org), masking the entire liver, and an erosion was applied to the manual mask prior to use in MRE inversion. Masks were drawn by one trained rater (D.W.) and checked and updated by a second (B.P.S.). Masks were drawn to include the entire liver, avoiding the edges of the liver, large arteries, or any visible image artifacts. MRE inversion for stiffness and damping ratio was performed using custom MATLAB code implementing Direct Inversion (DI) to produce the complex shear modulus, *G* = *G*′ + i*G*″, where *G*′ is the storage modulus and *G*″ is the loss modulus [17,18]. From the complex shear modulus, stiffness was calculated [19,20] as μ = 2|*G*|^2^/(*G*′ + |*G|*), and the damping ratio [21] as *ξ* = *G*″/2*G*′. For each, the pixel-by-pixel map was calculated, and the mean was taken over the eroded liver mask. Stiffness is given as mean ± standard deviation over multiple runs of MRE during the protocol.

### 2.4. Liver Biopsy and Tissue Processing

Liver tissue was procured at time points analogous to MRI/MRE, using liver biopsy at 1 and 2 months after liver fibrosis induction, and planned euthanasia with liver harvest at 3 months. Ultrasound-guided biopsies were performed the day after MRI/MRE scanning under general anesthesia. An 18-gauge device (BioPince; Argon Medical Devices, Plano, TX, USA) was advanced into the liver parenchyma. Core-needle samples (3–4) were obtained and saved in 10% neutral buffered formalin. Larger (20–30 g) right-lobe liver tissue samples were harvested from each Oncopig at the time of sacrifice. Formalin-fixed liver samples were then embedded in paraffin, sectioned at 5 μm, and mounted onto glass slides. Liver samples were stained with hematoxylin and eosin (H&E) and Masson’s trichrome. Specimens were blindly assessed by a board-certified veterinary pathologist and staged for fibrosis classification according to a porcine-adapted meta-analysis of histological data in the viral hepatitis (METAVIR) system (Table 2) [8]. An age-matched liver specimen from a 4-month-old Oncopig stained with H&E and Masson’s trichrome was used as a normal control.

### 2.5. Definitions, Outcome Measures, and Statistics

The primary outcome measure of this study was the capability to detect and quantify liver stiffness/fibrosis over time for individual Oncopig subjects using MRE, with radiologic–pathologic correlation of MRE imaging features with liver tissue histology. The threshold liver stiffness measure for MRE liver fibrosis was defined as 3 kPa, based on the threshold clinical value used to diagnose advanced chronic liver disease [12]. Statistical analysis was performed using Microsoft Excel (Redmond, WA, USA). Descriptive statistics were used to summarize quantitative data. Comparative analysis was performed using the two-sided Student’s *t*-test. Pearson correlation analysis was performed to determine the association between MRE measured liver stiffness and METAVIR fibrosis scores. A *p*-value < 0.05 defined statistical significance.

## 3. Results

### 3.1. Induction of Liver Fibrosis in Oncopigs

**Figure 2:**
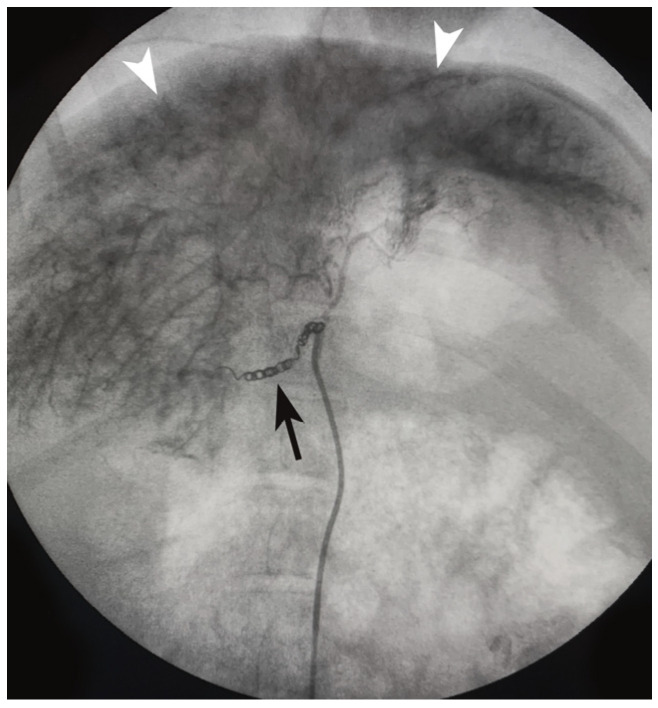
Fluoroscopic spot image demonstrates radiopaque ethanol-ethiodized oil staining of liver parenchyma (arrowheads) after fibrosis induction procedure (arrow denotes metallic coils in embolized gastroduodenal artery).

Liver fibrosis induction (Figure 2) and liver biopsies were technically successful in all cases. No immediate procedure-related adverse events (AEs) were observed. One delayed AE consisted of gastric ulceration and perforation, resulting in clinical signs and symptoms meeting humane endpoints and requiring euthanasia at 42 days following liver fibrosis induction.

**Figure 3:**
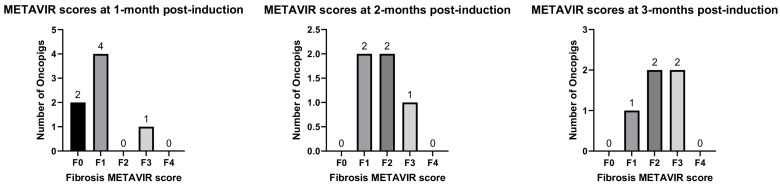
Graphical representation of liver fibrosis METAVIR outcomes at 1, 2, and 3 months post induction.

**Figure 4:**
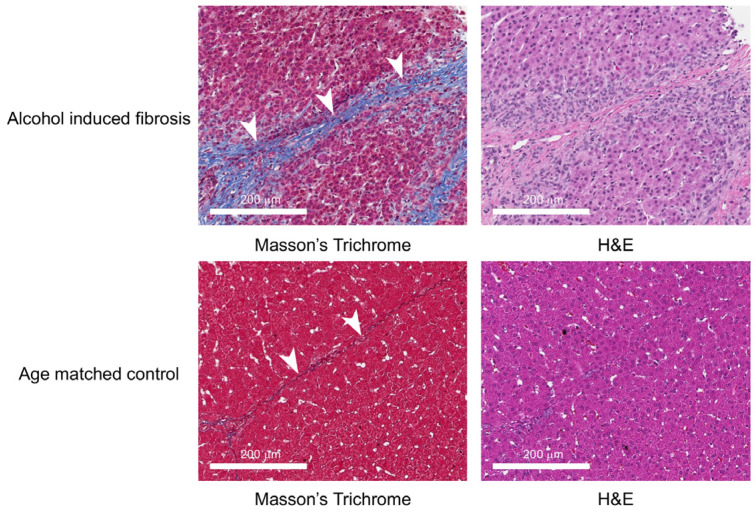
Representative Masson’s trichrome and H&E staining of Oncopig liver tissue 1 month after liver fibrosis induction, representing METAVIR F3 fibrosis, characterized by moderate-to-marked fibrous expansion of fibrous septa (arrowheads). Masson’s trichrome and H&E-stained specimens from age-matched control Oncopig liver illustrate normal porcine liver with thin fibrous septa separating hepatic lobules (arrowheads).

Liver fibrosis outcomes based on the METAVIR scoring of liver biopsies and tissues are depicted in Figure 3, and the METAVIR fibrosis scores for individual porcine subjects are summarized in Table 3. In fibrotic livers, thicker collagen bands were observed in Masson’s trichrome-stained specimens, while the age-matched control tissue had thinner collagen bands (Figure 4). Liver fibrosis was noted in 71% (5/7) subjects 1 month after fibrosis induction. The METAVIR fibrosis stages were the following: F0 = 2, F1 = 4, F3 = 1. At 2 months post liver fibrosis induction, parenchymal fibrosis was evident in 100% (5/5) of subjects with available liver tissue (1 animal was euthanized earlier for gastric ulceration and perforation, and liver tissue was not collected, while another tissue sample was inadvertently not processed for analysis). The METAVIR fibrosis stages at 2 months post liver fibrosis were F1 = 2, F2 = 2, F3 = 1. At 3 months post liver fibrosis induction, parenchymal fibrosis was evident in 100% (5/5) of subjects with available tissue samples. The METAVIR fibrosis stages at 3 months post liver fibrosis were F1 = 1, F2 = 2, F3 = 2.

### 3.2. Detection of Oncopig Liver Fibrosis Using MRE

Liver MRI/MRE was technically successful in all the attempted imaging sessions. A total of 7 out of 7 (100%) Oncopigs underwent 1-month MRI/MRE, 6/7 (86%) Oncopigs underwent 2-month MRI/MRE, and 5/7 (71%) Oncopigs underwent 3-month MRI/MRE (Figure 1). One animal subject was not imaged at 2 months or 3 months due to earlier euthanasia for gastric ulceration and perforation. Another animal subject was euthanized early due to budget constraints and did not undergo 3-month imaging. The MRI sequences depicted the anatomic and vascular structure of the liver with typical parenchymal signal intensities.

**Figure 5:**
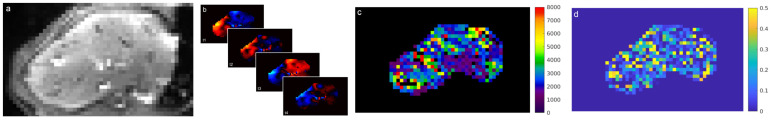
Liver MRE performed 1 month post liver fibrosis induction in Oncopig depicted in Figure 4. (**a**) Magnitude image from MRE acquisition. (**b**) Four time offsets for MRE actuation at 60 Hz with displacement encoding in z-direction (plus/minus subtracted). (**c**) Resulting stiffness map (μ, color scale in Pascals), and (**d**) damping ratio map (*ξ*, color scale unitless).

MRE successfully depicted liver fibrosis in the Oncopigs (Figure 5). MRE fibrosis (liver stiffness exceeding 3 kPa) was evident in 57% (4/7) of subjects at 1 month post fibrosis induction, with a mean liver stiffness of 2.94 ± 0.29 (range 2.41–3.40) kPa. At 2 months post liver fibrosis induction, parenchymal fibrosis was evident in 50% (3/6) of subjects, with a mean liver stiffness of 3.25 ± 0.43 (range 2.53–4.10) kPa. At 3 months post liver fibrosis induction, parenchymal fibrosis was evident in 40% (2/5) of subjects, with a mean liver stiffness of 2.91 ± 0.28 (range 2.21–3.32) kPa. Mean liver stiffness measurements were not significantly different over time (1-month vs. 2-month, *p* = 0.560; 1-month vs. 3-month, *p* = 0.886; 2-month vs. 3-month, *p* = 0.557). Liver stiffness measurements for individual Oncopigs are displayed in Table 4. The damping ratios, which quantify how much MRE oscillations are dampened out in tissue and have been used to detect hepatic inflammation [22], were 0.19 ± 0.05, 0.19 ± 0.04, and 0.18 ± 0.04 at 1, 2, and 3 months, respectively.

### 3.3. Correlation between Liver Imaging and Histology

The correlation analysis results indicated that the liver stiffness measurements and the METAVIR fibrosis scores did not significantly correlate (r = 0.056, *p* = 0.905 at 1 month; r = –0.076, *p* = 0.904 at 2 months; r = –0.534, *p* = 0.354 at 3 months). The liver stiffness measurements and damping ratios did not significantly correlate (r = 0.139, *p* = 0.767 at 1 month; r = –0.388, *p* = 0.447 at 2 months; r = 0.284, *p* = 0.644 at 3 months).

## 4. Discussion

In this small pilot study, MRI/MRE imaging was successfully performed and used to non-invasively detect and quantify liver fibrosis induced in Oncopigs. MRI offers the benefits of high-resolution soft tissue imaging, and the results of this work support the capability to develop advanced imaging techniques that are consistent and reproducible and offer meaningful diagnostic information for large-animal models of liver disease. For preclinical platforms like the Oncopig, non-invasive MRE can supersede liver biopsy, enhancing model safety and avoiding the bleeding risks associated with invasive diagnostic methods. Moreover, this approach provides a more global assessment of liver fibrosis by imaging the whole liver rather than relying on focal tissue sampling, which offers information about a single disease site as a surrogate for the whole organ. For this reason, measured liver stiffness did not correlate significantly with histological liver fibrosis stage; this was likely due to the small sample size used in this study and/or the less specific whole-liver MRE regions of interest vs. focal liver biopsies in the setting of fibrosis heterogeneity. In addition, biopsies were not MRI-guided, making co-registration not possible. It is also possible that MRE is less sensitive than tissue histology. Finally, the heterogeneity of histologic fibrosis could contribute to the lack of correlation. Potential minor disadvantages of MRI include the potential for lengthy imaging times, the need for general anesthesia to control animal subject respiration (though anesthesia is also required for liver biopsy), and the need for access to a scanner for veterinary imaging. In this study, a clinical MRI scanner (Siemens 3T Prisma) was utilized, but it was in a research setting, allowing for the transport and handling of the large Oncopig model. Overall, however, the results of this study support the potential utility of MRE in confirming liver fibrosis in the Oncopig model of liver disease.

There have been few prior publications evaluating the use of MRE for the detection of liver fibrosis in in vitro and in vivo porcine livers. In 2017, Yang et al. studied six explanted pig livers imaged with MRE prior to and following treatment with 10% neutral buffered formalin for 3–4 days [23]. Their major outcomes included an approximately three-fold increase in MRE-measured liver stiffness after treatment with formalin [23], which can change hepatic structural properties. The study confirmed the capability of MRE to non-invasively quantify alterations in liver stiffness. In 2013, Huang et al. studied the capability of MRE to detect in vivo liver fibrosis in eight pigs that underwent transarterial ethanol-ethiodized oil-induced liver fibrosis [24]. In that study, the animal subjects underwent MRE and tissue harvest 4 weeks after liver fibrosis induction [24]. Their principal findings included an increase in mean MRE-measured liver stiffness by 0.82 kPa, a mean METAVIR fibrosis grade of 2.8, and a positive correlation between liver stiffness and liver fibrosis (ρ  =  0.884; *p* < 0.001) [24]. It is unclear whether the reported use of the most severely affected regions of the liver for histologic grading of fibrosis could have potentially influenced the observed results of the correlation analysis in contrast to the results of this investigation. In 2017, Yin et al. utilized a fumarylacetoacetate hydrolase-deficient pig model for monthly liver MRE imaging for more than 1 year, demonstrating increasing shear stiffness over time in two animal subjects [22]. The findings of the investigation supported the capability of MRE for longer-term liver imaging and fibrosis tracking.

The Oncopig, capable of inducible site- and cell-specific tumor generation, is a useful model for the preclinical investigation of liver cancer and associated comorbid liver fibrosis, using the same tools used in human clinical practice. The ability to develop METAVIR F2-F4 alcohol-induced liver fibrosis in the Oncopig has previously been demonstrated [8,9,10], providing a vehicle to study HCC development in a cirrhotic liver background. With these attributes, the Oncopig has the potential to serve as a valuable transitional bridge between preclinical small-animal murine studies and human clinical trials. As a model of disease, the Oncopig can undergo human-scale imaging—which can be correlated with tissue to validate findings—across multiple time points. The long lifespan of the Oncopig permits long-term systematic imaging to support longitudinal studies across disease states, which can facilitate studies aimed at prognostic assessments and therapeutic response evaluation.

The development and preclinical validation of an MRI/MRE protocol for the detection, diagnosis, characterization, and quantification of liver fibrosis in the Oncopig large-animal model using advanced non-invasive radiologic imaging has critical importance given the central role of radiologic imaging in clinical diagnostics, treatment, and follow-up. For clinical practice, MRE has been shown to have excellent accuracy in the detection of liver fibrosis across liver disease etiologies [25] and can be used in various applications spanning disease diagnosis, etiology distinction, staging, longitudinal monitoring, treatment response assessment, and outcome prediction [26]. As such, the development and application of a minimally invasive MRI/MRE liver imaging protocol tailored for large-animal imaging can facilitate preclinical trials using qualified large-animal models—such as the Oncopig—aimed at the discovery and therapeutic response monitoring of liver disease in order to undertake translational investigations aimed at significantly improving prevention, detection, and survival in patients with these conditions. Without enabling such technologies for preclinical investigation and clinical translation, pathways toward the early detection, diagnosis, and treatment of liver disease are less likely.

This study had limitations. First, the sample size was small, though this investigation was intentionally designed as a pilot study to investigate the feasibility of MRE for the non-invasive diagnosis of Oncopig liver fibrosis. Second, there was suboptimal registration between MRE and liver biopsies, though this may in fact reflect an innate limitation of focal liver biopsy as an indicator of whole-liver parenchymal disease. Third, Oncopig subjects underwent only one liver fibrosis induction procedure, which limits the disease severity, and liver fibrosis induced in this manner may resolve over time [8]. A protocol of multiple transarterial procedures and/or daily oral alcohol intake for more consistent alcohol exposure may better mirror advanced chronic liver disease. Fourth, the metallic coils used to embolize the gastroduodenal artery resulted in some magnetic susceptibility artifacts on the EPI-based sequences used to complete the MRE, which limited stiffness quantification in the immediately adjacent liver parenchyma. Fourth, this study did not employ other imaging techniques like transient elastography, which could be used to quantify liver fibrosis. Rather, MRI/MRE was selected due to its advantages, including excellent soft tissue anatomic depiction, which not only offers potential benefits in the diagnosis and staging of liver fibrosis but may also be used to diagnose and follow liver tumors that are created in the Oncopig. Fifth, there were limited data from control subjects with which to compare outcomes in subjects with liver fibrosis.

## 5. Conclusions

In conclusion, MRE-quantifiable liver fibrosis may be induced in the Oncopig, and the results presented herein support the potential utility of MRE in non-invasively detecting liver fibrosis. However, the measured liver stiffness did not correlate significantly with the histological liver fibrosis stage, which may be due to the small sample size and/or misregistration between MRE regions of interest and liver biopsies. Future studies may focus on coordinating elastography maps with liver biopsy location to better reflect the association between stiffness and fibrosis and may also incorporate methods of chronic fibrosis induction.

## Figures and Tables

**Table 1:** MRI/MRE acquisition protocol.

Sequence	Respiratory Gating	Purpose	Main Parameters
T2 BLADE with fat saturation, transversal	Imaging-based	T2-weighted images	TE/TR 144/2500 ms, 38 cm FOV, 20 slices (5 mm skip 25%), voxel size 1.2 × 1.2 × 5 mm, 15% accept window
T2 BLADE without fat saturation, transversal	Imaging-based	T2-weighted images with fat included	TE/TR 156/2500 ms, 38 cm FOV, 20 slices (5 mm skip 25%), voxel size 1.2 × 1.2 × 5 mm, 15% accept window
TSE Dixon, transversal	Imaging-based	Fat/water images; highlight fatty tumors	TE/TR 99/4480 ms, 34 cm FOV, 35 slices (5 mm skip 20%), voxel size 0.9 × 0.9 × 5 mm, 15% accept window
TurboFLASH in-phase/opposite-phase images, transversal	Imaging-based	In-phase and opposed-phase images for fat/water to show fatty infiltration	TE/TR 2.46/1800 ms, 38 cm FOV, 20 slices (5 mm skip 25%), voxel size 0.7 × 0.7 × 5 mm, 15% accept window
T1 TurboFLASH, coronal	Imaging-based	T1-weighted images	TE/TR 2.31/2000 ms, 40 cm FOV, 30 slices (5 mm skip 20%), voxel size 0.8 × 0.8 × 5 mm, 15% accept window
Pd + T2 + T2 TSE, transversal	Imaging-based	Multiple contrast from proton density to T2 to TSE T2	TE1/TE2/TE3/TR 41/107/205/2500 ms, 20 slices (5 mm skip 25%), voxel size 1.5 × 1.5 × 5 mm, 15% accept window
EPI diffusion-weighted imaging, transversal	No gating	Diffusion-weighted images for Trace with b-values of 50 and 800 s/mm^2^	4 scan trace, TE/TR 39/4900 ms, 36 slices (4 mm skip 20%), voxel size 1.4 × 1.4 × 4
EPI MRE, transversal	Breath hold	Elastography	TE/TR 35/1200.96 ms, 12 slices (5 mm skip 0%), voxel size 4.9 × 4.9 × 5 mm, 60 Hz, 4 time points, 8.927 μm/rad, duration 34 s
T1 VIBE, transversal	Breath hold	DCE venous enhancement characteristics	TE1/TE2/TR 1.29/2.52/3.97 ms, 1 slice 3 mm, voxel size 1.2 × 1.2 × 3 mm, duration 15 s Run at pre injection (x2), then post injection at 20 s, 50 s, 2 min, 5 min, and 7.5 min

MRI = magnetic resonance imaging, MRE = magnetic resonance elastography, BLADE = balanced steady-state free precession line acquisition with undersampling, FOV = field of view, FLASH = fast low-angle shot, TSE = turbo spin echo, EPI = echo-planar imaging, VIBE = volumetric interpolated breath-hold examination, DCE = dynamic contrast-enhanced, TE = time to echo, TR = repetition time.

**Table 2:** Porcine-adapted METAVIR fibrosis scoring system.

Grade	Description
F0	Normal porcine liver; no increase in fibrosis.
F1	Mild fibrous expansion of portal areas and/or mild thickening/expansion of few random segments of normal pre-existing fibrous septa.
F2	Mild-to-moderate fibrous expansion of portal tracts and multiple, random, non-contiguous segments of normal fibrous septa surrounding multiple hepatic lobules ± presence of thin bands of fibrosis extending from septa or portal tracts into adjacent lobular parenchyma.
F3	Moderate-to-marked fibrous expansion of contiguous segments of fibrous septa surrounding multiple hepatic lobules; fibrous expansion can involve contiguous segments of septa and partially encircle hepatic lobules, but it typically does not completely circumscribe lobules. Presence of fibrous connective tissue that dissects into lobular parenchyma, surrounding and separating cords of hepatocytes.
F4	Cirrhosis; normal fibrous septa surrounding hepatic lobules are expanded by moderate-to-marked amounts of fibrous connective tissue, with some portal bridging, frequent dissection into adjacent lobular parenchyma, and separation of hepatic cords. Fibrous connective tissue often completely circumscribes multiple hepatic lobules, which appear irregular/shrunken.

METAVIR = meta-analysis of histological data in viral hepatitis.

**Table 3:** METAVIR fibrosis scores for individual porcine subjects.

Pig	1 Month	2 Months	3 Months
A337	F1	F1	F2
A339	F1	F2	F3
A341	F1	F1	F3
A342	F0	F2	F2
A344	F3	-	-
A345	F1	F3	F1
A347	F0	-	-

METAVIR = meta-analysis of histological data in viral hepatitis.

**Table 4:** MRE-measured liver stiffness (mean ± SD) for individual Oncopigs over time.

Pig	1-Month Liver Stiffness (kPA)	2-Month Liver Stiffness (kPA)	3-Month Liver Stiffness (kPA)
A337	2.51 ± 0.43	2.53 *	2.90 ± 0.52
A339	2.41 ± 0.29	2.76 ± 0.65	2.21 ± 0.15
A341	2.80 ± 0.26	4.10 ± 0.77	3.23 ± 0.36
A342	3.07 ± 0.51	2.83 ± 0.76	2.87 ± 0.71
A344	3.30 ± 0.24	-	-
A345	3.11 ± 0.11	3.36 ± 0.71	3.32 ± 0.32
A347	3.40 ± 0.18	3.94 ± 0.32	-

MRE = magnetic resonance elastography, SD = standard deviation. * Stiffness is given as the mean ± SD over multiple runs of MRE during the protocol. For Pig A337, only one run was completed at the 2-month time point.

## Data Availability

Data may be made available upon request.
